# Editorial: Mental health literacy: How to obtain and maintain positive mental health

**DOI:** 10.3389/fpsyg.2022.1036983

**Published:** 2022-10-12

**Authors:** Carlos Sequeira, Francisco Sampaio, Lara Guedes de Pinho, Odete Araújo, Teresa Lluch Canut, Lia Sousa

**Affiliations:** ^1^Nursing School of Porto, Porto, Portugal; ^2^CINTESIS@RISE, Nursing School of Porto (ESEP), Porto, Portugal; ^3^Higher School of Health Fernando Pessoa, Porto, Portugal; ^4^Nursing Department, University of Évora, Évora, Portugal; ^5^Comprehensive Health Research Centre, University of Évora, Évora, Portugal; ^6^School of Nursing, University of Minho, Braga, Portugal; ^7^Health Sciences Research Unit: Nursing (UICISA:E), Nursing School of Coimbra (ESEnfC), Coimbra, Portugal; ^8^Nursing Research Centre, University of Minho, Braga, Portugal; ^9^Department of Public Health, Mental Health, and Maternal and Child Health Nursing, Faculty of Medicine and Health Sciences, University of Barcelona, Barcelona, Spain; ^10^Vale do Ave Higher School of Health, Vila Nova de Famalicão, Portugal

**Keywords:** mental health, health literacy, health promotion, sense of coherence, self-care

There is a growing consensus within the scientific community about the importance of mental health promotion. Around one billion people worldwide have a mental disorder, and anyone, anywhere, can be affected (World Health Organization, 2022). It is estimated that one in four people has some mental health disorder. It is widely known that the incidence and prevalence of mental disorders are increasing, on the one hand, due to the social pressure exerted by current lifestyles and, on the other hand, because people do not always have emotional regulation mechanisms and resilience that allow them to adaptively deal with adverse life events.

The COVID-19 pandemic has exposed and included many of these problems on the agenda, although they are not new. The persistent low funding for mental health services hinders access to mental health promotion, prevention, and treatment of mental disorders. In low- and middle-income countries, more than 75% of people with mental health problems do not receive any treatment at all, and the economic investment of countries in the mental health of their populations remains scarce (World Health Organization, 2022).

Mental health promotion must be based on the identification of existing personal, social, environmental and cultural determinants to enable the implementation of measures to mitigate these factors and develop protective factors in mental health, such as resilience and the existence of environments (schools, workplaces, among others) to support mental health. These interventions can be developed in different contexts, in groups or individually. Mental health promotion and prevention programs must transcend the health sector and involve sectors such as education, work, the environment, and housing, among others (World Health Organization, 2022). Currently, the main priorities in mental health promotion are children and young people, suicide prevention, and mental health promotion in the workplace.

When referring to mental health promotion, one of its intrinsic variables is mental health literacy. Mental health literacy is the knowledge and beliefs about mental disorders which aid their recognition, management, or prevention (Jorm, 2000). Mental health literacy encompasses essentially four components: understanding how to achieve and maintain good mental health, understanding mental disorders and their treatments, decreasing the stigma related to mental disorders, and increasing the effectiveness of help-seeking (Nobre et al., 2021). However, some authors include optimism and hope as some of the predictors of mental health, as well as creativity.

Positive mental health literacy is considered a component of mental health literacy, which needs clarification and attention throughout the lifespan in different contexts and intervention levels. Positive mental health has some important effects and can be adopted in psychiatric contexts, improving patients' quality of life and preventing psychotic outbreaks.

Mental health literacy is the first step toward mental health promotion and an essential component for the good mental health of populations. The WHO, in its “Mental Health Action Plan 2013–2020,” stated that mental health literacy is one of the strategies to be used to promote and prevent mental health problems (World Health Organization, 2013). However, although evidence shows that the level of mental health literacy in the general population has been progressively increasing, it is still low/moderate (Nobre et al., 2021).

Thus, developing mental health literacy in the general population, particularly in some settings and of some priority groups, such as adolescents, in the workplace, and family caregivers, is fundamental to developing concerted strategies to promote mental health.

The mental health challenges throughout the life cycle are well known. In the infancy stage, educators have an essential role in providing psychosocial support and several measures need to be developed to face the challenges. However, adolescents may be at risk of mental health problems due to their environments, including discrimination, poverty, abuse or violence, and lack of access to adequate support. According to the World Health Organization (2018), it has been estimated that about 10–20% of adolescents have already experienced mental health problems. In addition, some problems were aggravated by the COVID-19 pandemic (e.g., anxiety or addictions), especially in university settings. Regarding this topic, some evidence suggests that proper intervention in physical activity and some mental health strategies are beneficial to reducing anxiety in academic students. Resilience also plays an essential role in the mental health of college students. Furthermore, some variables (e.g., grit) were revealed to be predictors of greater career adaptability through greater career exploration and decision-making self-efficacy, positive affect, and commitment to goals. The results of this study, carried out in China with 839 Chinese college students, open the way for other studies, especially in academic contexts.

The existence of validated assessment tools that measure mental health literacy and help-seeking behavior can also support professionals in measuring this phenomenon (firstly) and to intervening properly. These concerns should be addressed when there are mental health problems in adolescence to avoid more severe mental health problems in adulthood.

In addition, it is crucial that parents, teachers, and the whole school community develop sufficient knowledge and skills about mental health for the early identification of mental health problems in young people. Recognizing changes in physical and mental health can support those closest to them in making referrals for specialist help. In recent years, educational programs aimed at parents have been developed, although more evidence is needed to determine the effectiveness of these programs in enabling further dissemination and prevention of mental disorders in young people. There is a clear idea that more programs which promote mental health literacy are crucial for parents of adolescents. Similarly, mental health literacy programs involving the school community (teachers, operational assistants, students) should be further investigated. Teachers' social support is associated with their mental health literacy, coping tendency, and life satisfaction; coping tendency is associated with mental health literacy and life satisfaction; and life satisfaction is associated with mental health literacy.

Only in an integrated and articulated way will it be possible to improve mental health literacy and substantially decrease the risk of mental disorders in adulthood. Some studies revealed that young people expressed concerns about the lack of mental health education in their schools and indicated that this must change for the wellbeing of all youth.

The importance and relationship between work (values) and mental health and its negative consequences have been the subject of intense debate. Some studies show that positive work values can promote life satisfaction and seem to be a protective factor for mental health. Promoting mental health literacy in work contexts means empowering work partners to promote mental health-promoting environments and recognizing the importance of identifying risk situations that lead to loss of health and consequent mental disorders, with serious implications for the individual, companies and society as a whole. Workplace mental health literacy is also vital in self-stigma promotion.

Nevertheless, other studies suggested that teachers generally suffer from job burnout, and their personality characteristics have a significant impact on it. School managers should pay particular attention to this problem and implement necessary interventions.

Currently, the increasingly technological challenges are prompting their use and adaptation to new interventions in clinical practice. E-learning can provide broad access in various settings allowing for new learning paths and adapted training paces.

Regarding family caregivers, some important issues must be considered. The experience of taking care is physically and emotionally demanding due to the previous relationships and the type and duration of the provided care. Evidence has been particularly compelling about the importance of the caregivers' mental health when caring for a relative; however, there is still little research on the post-caregiver experience. This means that caring for someone presupposes a transition that often leads to the reconstruction of a new identity that starts before the relative's death and continues beyond the grieving process. Because caregivers have ongoing emotional needs, post-care indicates that this stage should be considered part of the caregiver's life. Future studies should focus on developing structured interventions to promote the caregivers' mental health and support other caregivers going through the same life experience (linked to positive mental health factors).

The articles which are part of this Research Topic highlighted the need to consider mental health literacy as a priority, aiming not only for the near future but also for the present. Thus, they contributed to a better understanding of the state of the art on this topic.

Now, it is crucial to move on from opinion papers and observational studies to experimental research. The needs, mainly the ones of children, a, and young adults, are clearly identified, and they tend to be consensual: there is a lack of mental health literacy, even though the COVID-19 pandemic raised the discussion on mental health issues. Thus, it is mandatory to develop some mental health literacy promotion interventions and evaluate their efficacy, effectiveness, and cost-effectiveness. This would reinforce the research community's commitment to the goal of developing innovative solutions to improve the mental health literacy of the population.

Nonetheless, not only the research community should be committed to this topic, but the governments and decision-makers also play a key role in this strategic mission. It is widely known that some political decisions have a significant (indirect) impact on people's mental health. Therefore, political decisions that can protect people's mental health are currently an unquestionable requirement. One of those potential decisions is to include content about mental health issues (not only about mental disorders) in the school curricula. Also, it is important to assign a mental health nurse and/or a psychologist to every school, as they can help children and/or adolescents to cope with negative life events, early recognize potential mental health problems, and improve their help-seeking behavior.

A serious investment in mental health literacy promotion has significant costs; however, it will surely help reduce, for example, psychiatric hospitalizations in the future. That is not only a social benefit but also an economic one. Scientific evidence on this topic has been published in several journals, including this Research Topic; now, it is time to put knowledge into practice.

## Author contributions

All authors listed have made a substantial, direct, and intellectual contribution to the work and approved it for publication.

## Funding

This work was financed by national funds through FCT Fundação para a Ciência e a Tecnologia, I.P., within the scope of the project RISE - LA/P/0053/2020.

## Conflict of interest

The authors declare that the research was conducted in the absence of any commercial or financial relationships that could be construed as a potential conflict of interest.

## Publisher's note

All claims expressed in this article are solely those of the authors and do not necessarily represent those of their affiliated organizations, or those of the publisher, the editors and the reviewers. Any product that may be evaluated in this article, or claim that may be made by its manufacturer, is not guaranteed or endorsed by the publisher.
